# GABA-B Controls Persistent Na^+^ Current and Coupled Na^+^-Activated K^+^ Current

**DOI:** 10.1523/ENEURO.0114-17.2017

**Published:** 2017-06-23

**Authors:** Ping Li, Richard Stewart, Alice Butler, Ana Laura Gonzalez-Cota, Steve Harmon, Lawrence Salkoff

**Affiliations:** 1 Department of Neuroscience, Washington University School of Medicine, St. Louis, MO 63110; 2 Department of Genetics, Washington University School of Medicine, St. Louis, MO 63110

**Keywords:** Baclofen, GABA-B, olfactory bulb, persistent sodium current, potassium channel, SLO2, mitral cell, Slick

## Abstract

The GABA-B receptor is densely expressed throughout the brain and has been implicated in many CNS functions and disorders, including addiction, epilepsy, spasticity, schizophrenia, anxiety, cognitive deficits, and depression, as well as various aspects of nervous system development. How one GABA-B receptor is involved in so many aspects of CNS function remains unanswered. Activation of GABA-B receptors is normally thought to produce inhibitory responses in the nervous system, but puzzling contradictory responses exist. Here we report that in rat mitral cells of the olfactory bulb, GABA-B receptor activation inhibits both the persistent sodium current (INa_P_) and the sodium-activated potassium current (IK_Na_), which is coupled to it. We find that the primary effect of GABA-B activation is to inhibit INa_P_, which has the secondary effect of inhibiting IK_Na_ because of its dependence on persistent sodium entry for activation. This can have either a net excitatory or inhibitory effect depending on the balance of INa_P_/IK_Na_ currents in neurons. In the olfactory bulb, the cell bodies of mitral cells are densely packed with sodium-activated potassium channels. These channels produce a large IK_Na_ which, if constitutively active, would shunt any synaptic potentials traversing the soma before reaching the spike initiation zone. However, GABA-B receptor activation might have the net effect of reducing the IK_Na_ blocking effect, thus enhancing the effectiveness of synaptic potentials.

## Significance Statement

The GABA-B receptor is densely expressed in the brain and implicated in many CNS functions and disorders, but knowledge of its mode of action is limited. We have found a novel action whereby GABA-B receptor activity inhibits two opposing currents, the persistent sodium current and the sodium-activated potassium current that is activated by it. It is likely that GABA-B receptor activation through this coupled system of currents can have either a net excitatory or inhibitory response depending on the balance of currents. Extensive colocalization of GABA-B receptors and sodium-activated potassium channels throughout the nervous system suggests a significant mechanism for GABA-B neuromodulation, and our results suggests new insights for controlling cell excitability through GABA-B modulators.

## Introduction

γ-Amino butyric acid (GABA) is the major inhibitory neurotransmitter in the CNS. Two major classes of proteins are known that mediate GABA responses: the ionotropic GABA-A receptor family (a chloride ion channel) and the metabotropic GABA-B receptor. The GABA-B receptor is not a channel itself, but a G protein–coupled receptor (GPCR) that regulates various ion channels and other functions by G protein signaling. The GABA-B receptor has been implicated in many CNS functions and disorders (Bettler et al., 2004) and is also involved in various aspects of nervous system development (Prosser et al., 2001; Schuler et al., 2001). How this single GABA-B receptor induces so many varied downstream functions remains a major unanswered question. There are currently two well-characterized ion channel targets responsive to GABA-B signals: N-type calcium channels, which are typically presynaptic and inhibited by GABA-B signaling, and the GIRK family of inward-rectifying potassium channels, which are typically postsynaptic and activated by GABA-B signaling (Bettler et al., 2004). Both actions have a net inhibitory effect. However, unexplained contradictory excitable responses are also present that greatly limit the clinical usefulness of GABA-B activators such as baclofen (Vergnes et al., 1997; Bowery, 2006).

We now report a novel effect of GABA-B signaling on the INa_P_/IK_Na_ coupled system. We recently reported the existence of this unusual system in which persistent sodium current (INa_P_) activates high-conductance sodium-activated potassium currents (IK_Na_; Budelli et al., 2009; Hage and Salkoff, 2012). The components of this coupled system are widely distributed throughout the brain (Dryer, 1994; Kaczmarek, 2013) and control the electrical excitability of various neuronal types (Dryer, 1994; Bhattacharjee and Kaczmarek, 2005). INa_P_ enhances neuronal excitability, and blocking INa_P_ (without blocking the transient sodium current) is a common strategy used in designing pharmacological agents to prevent seizures (Rogawski and Loscher, 2004; Mantegazza et al., 2010). However, our results showed an unanticipated role for INa_P_ in activating sodium-activated potassium currents which act as a negative feedback system in countering its own excitable effects. Whether these two interacting currents have a net excitable or inhibitory effect depends on the amplitudes and balance of these two currents in a particular neuronal type. Our results now show that activation of the GABA-B pathway reduces both of these currents, INa_P_ directly and IK_Na_ indirectly as a secondary consequence of inhibiting the Na^+^ entry pathway that activates it.

This mechanism of GABA-B action may interface with other functions of GABA-B even in the same cell types. In the mammalian olfactory bulb, GABA-B receptors are present on the lateral dendrites and cell bodies of mitral cells. GABA is released at these sites from the synapses of dendrites of associated granule cells. These two cell types undergo complex dendrodendritic interactions which may involve several mechanisms of GABAergic signaling (Isaacson and Vitten, 2003).

## Materials and Methods

### Primary neuronal culture preparations

Primary cultures of dissociated olfactory bulbs of neonatal Sprague-Dawley rats of either sex were prepared following methods described previously (Trombley and Westbrook, 1990). In brief, dissected olfactory bulbs were incubated in 0.25% trypsin in PBS for 30 min at 37°C. For the last 5 min, trypsin solution was supplemented with 0.05% DNase. Cells were then triturated and plated on poly-lysine–coated glass coverslips in Dulbecco’s modified Eagle medium/F-12 media (Gibco) with 10% FBS and 2% B-27 supplement (Gibco). 3 d after plating, media was replaced with Neurobasal (Gibco) + 2% B-27 supplement and 5 μm arabinofuranosyl cytidine to inhibit division of glia. Whole-cell recordings were performed 3–6 d after plating.

### Primary neuronal culture immunostaining

Olfactory bulb neurons were cultured on 12-mm-diameter coverslips for 10–14 d *in vitro*. Cells were fixed with 4% paraformaldehyde in PBS for 15 min at 4°C and stored in PBS at 4°C for future use. Cells were incubated for 1 h in blocking solution [Tris-buffered saline (TBS) with 2% fetal bovine serum (Invitrogen)]. Cells were permeabilized with 0.2% saponin (Sigma-Aldrich) in TBS for 15 min at room temperature. Primary antibody incubations (2 μg/ml in blocking buffer) were performed either for 1 h at room temperature or overnight at 4°C; cells were washed for at least 30 min at room temperature with TBS. Secondary antibodies were incubated for 1 h at room temperature in blocking buffer, then washed as above. Cells were stained with Hoechst 33258 (Sigma-Aldrich) to identify nuclei and mounted on microscope slides in Fluoro-shield (Sigma-Aldrich). Cells were observed with a Nikon Eclipse E800 microscope and a 60× oil-immersion objective and imaged with a CoolSnap EZ CCD camera and Qcapture (QImaging). Only images from mitral/tufted cells (large neurons with a pyramidal morphology) are shown. SLO2.1 and NaV 1.6 immunostaining was abolished by preincubation with the antigenic peptide for 1 h at room temperature. The two different antibodies to SLO2.1 (also known as Slick) both produce highly similar results. Representative images from at least three separate neuronal preparations produced similar results.

Primary antibodies were purchased from the following sources: rabbit anti-NaV 1.6 (AB5580, EMD Millipore, 1:1000, RRID:AB_91916); rabbit anti-Slick (APC-126, Alomone labs, 1:500, RRID: AB_10613114); mouse anti-Slick (73-055, clone N11/33, NeuroMabs, 1:50, Antibody Registry AB_10675450); mouse anti-GABAB-R1 (73-183, clone N93A/49, NeuroMabs, 1:50, Antibody Registry AB_10672843). Fluorophore-coupled anti–mouse IgG and anti–rabbit IgG secondary antibodies were purchased from Sigma-Aldrich.

#### Xenopus oocytes injection

*Xenopus laevis* oocytes were injected with 50 nl cRNA (1–3 μg/μl) using a Drummond Scientific nanoinjector. Injected oocytes were incubated at 18°C in ND96 medium (in mM): 96 NaCl, 2 KCl, 1.8 CaCl_2_, 1 MgCl_2_, and 5 HEPES, pH 7.5, with NaOH. Electrophysiology experiments on oocytes were undertaken 3–5 d after injection.

#### Voltage-clamp recordings and analysis

Tufted/mitral cells were identified on the basis of their pyramidal shape and large size. Voltage-clamp recordings were performed using an Axopatch 200B amplifier (Molecular Devices). Recordings were filtered at 2 kHz with the internal filter of the amplifier and digitized at 10 kHz using a Digidata 1322A digitizer (Molecular Devices). Recording pipettes were pulled from borosilicate glass with tip resistances of 3–5 MΩ after filling with internal pipette solution contained (in mM): 140 KCl, 1 MgCl_2_, 10 HEPES, and 5 EGTA, pH 7.3, with KOH. Bath solutions contained (in mM): 150 NaCl, 5 KCl, 2 MgCl_2_, 10 HEPES, and 10 glucose, pH 7.3, with NaOH. For slow-ramp protocol recordings, the internal solution contained (in mM): 140 KMES, 1 MgCl_2_, 10 HEPES, and 5 EGTA, pH 7.3, with KOH. Tetrodotoxin (TTX), baclofen, and GABA were used at 0.2, 10, or 100 μM for ∼3 min before recording.

Two-electrode voltage-clamp experiments in *Xenopus* oocytes injected with SlO2.1 cRNA were undertaken in ND96 plus 1–2 mM DIDS to block the endogenous chloride conductances. Voltage steps (−80 to 40 mV) were applied in 10-mV increments from a holding potential of −70 mV. Recording pipettes were filled with 3 M KCl. Baclofen was applied to the recording chamber by continuous perfusion. TTX, baclofen, and GABA were prepared daily from 3-, 5-, or 500-mM stock solutions (in ddH_2_O). All chemicals were purchased from Sigma-Aldrich.

Whole-cell recordings were acquired and analyzed with pClamp 9.0 (Molecular Devices). Statistical analysis was performed using previously described tools (Budelli et al., 2009; Hage and Salkoff, 2012). For comparison of results between two groups, paired Student’s *t* tests were used for the same procedures before and after applied treatments. One-way ANOVA was used to determine whether there were statistically significant differences among the effects of the three drugs (TTX, baclofen, and GABA).

## Results

**Figure 1.**
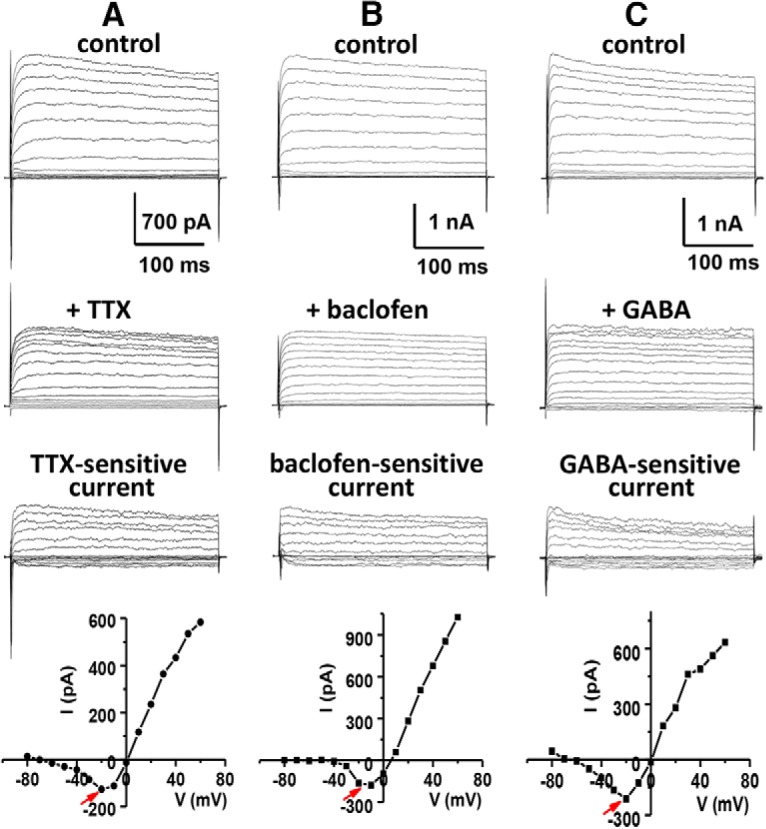
TTX, baclofen, and GABA all inhibit inward and outward currents. Representative traces of whole-cell currents recorded from cultured rat mitral cells in the absence and presence of 0.2 µM TTX (***A***), 10 µM baclofen (***B***), or 100 µM GABA (***C***). The top traces show the control currents evoked from a holding potential of –70 mV in steps from –80 to 60 mV in 10-mV intervals. The middle traces show the residual currents after application of TTX (***A***), baclofen (***B***), or GABA (***C***). The bottom traces show TTX-sensitive (***A***), baclofen-sensitive (***B***), or GABA-sensitive (***C***) current that is obtained by subtraction of the residual current (after drug application) from the control current (before drug application). After application of TTX, baclofen, or GABA, the outward current reduction at 40 mV was 26.8 ± 2.4% (*n* = 12, *p* < 0.0001), 30.4 ± 2.5% (*n* = 12, *p* < 0.0001), or 22.9 ± 2.1% (*n* = 5, *p* < 0.0001), respectively. The I-V plot determined from steady-state values of drug-sensitive current is given below each group of sample traces. The removed inward component of current is indicated by an arrow. Note that the current/voltage plots below the current traces indicate steady state current measured ∼250 ms after the initiation of the step pulses. Because of the overlap of rapidly activating inward and outward currents at the initiation of the step pulses, the currents shown in the first few milliseconds of the traces may not be an accurate depiction of their kinetic properties.

Mitral cells, the main output neurons of the olfactory bulb, are known to express a large component of sodium-activated potassium current (IK_Na_) (Egan et al., 1992a, b; Bhattacharjee and Kaczmarek, 2005). We previously showed that IK_Na_ is activated by sodium entry through the persistent current component of voltage-dependent sodium channels (Hage and Salkoff, 2012). As with the transient component of sodium current, the persistent current component can also be blocked by the sodium channel blocker TTX. Application of TTX to a voltage-clamped neuron results in the removal of a large non-inactivating outward current corresponding to IK_Na_ (Fig. 1*A*). To support the hypothesis that the TTX-sensitive outward current is a sodium-dependent current, we produced virtually identical results by replacing extracellular sodium ion with either choline or lithium ion (Budelli et al., 2009). Both techniques removed the same outward current component. Our previous study also revealed that a similar sodium-dependent outward current is present in several neuronal types and that the current is carried by the SLO2 family of high-conductance potassium channels (Budelli et al., 2009; Hage and Salkoff, 2012). The removal of this outward current by blocking sodium channels or by eliminating sodium ions showed that it was being removed as a secondary consequence of the elimination of a sodium current; we showed this to be the persistent component of the sodium current (Hage and Salkoff, 2012). Fig. 1*A* shows whole-cell currents recorded from a cultured rat mitral cell before (top traces) and after (middle traces) application of 0.2 μM TTX. After application of TTX, the reduction in outward current at 40 mV was 26.8 ± 2.4% (*n* = 12, *p* < 0.0001). The TTX-sensitive IK_Na_, which decays only slightly over a time course of a second, is revealed by subtraction of TTX-resistant current from control current (bottom traces). The bottom panel of Fig. 1*A* shows the current–voltage relationship of the TTX-sensitive current. It should be noted that there is an inward current component (arrow) preceding the outward current in this TTX-sensitive I-V plot. Previously we showed that this inward component represents a TTX-sensitive persistent sodium current that is active over a wide voltage range (Hage and Salkoff, 2012). Note that the inward currents indicated by arrows in the bottom panels of Fig. 1 represent steady-state persistent currents that were plotted at a time point long after the inactivation of the transient component of sodium conductance (see legend). However, both the transient and persistent components of sodium current in mitral cells are blocked by TTX (Hage and Salkoff, 2012).

### Neuromodulation of sodium-activated K^+^ currents in native cells

**Figure 2.**
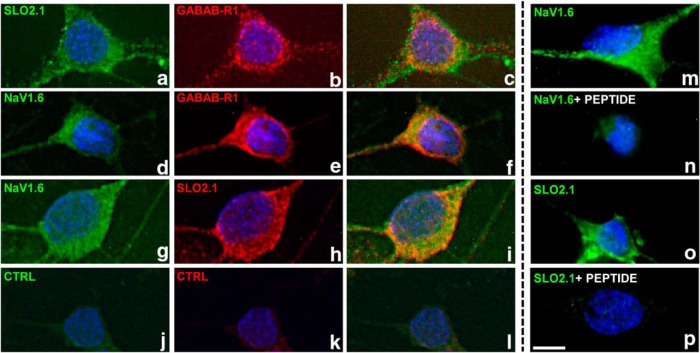
The GABA-B receptor (GABAB-R1) colocalizes with sodium-activated potassium channels (SLO2.1) and voltage-sensitive sodium channels (NaV1.6). Rat olfactory bulb primary neurons were immunostained with pairwise combinations of primary antibodies (indicated at top left) raised from different species, followed by staining with fluorophore-coupled secondary antibodies. Panels ***c***, ***f***, and ***i*** are merged images, demonstrating that both proteins are present in the same cell. As with other channels and receptors, there is likely an intracellular pool as well a cell-surface pool, consistent with previous results (Panzanelli et al., 2004). Control samples incubated without any primary antibody show negligible staining (panels ***j***, ***k***, and ***l***). The last column shows additional examples of immunostaining for SLO2.1 or NaV 1.6 in the absence or presence of the antigenic peptide (panels ***m***, ***n***, ***o***, and ***p***); the immunostaining is dramatically reduced in the presence of the peptide. Scale bar in ***p***: 5 μm.

One distinguishing structural feature of SLO2 channels that carry the sodium-activated K^+^ current is the absence of a string of positively charged amino acids in the S4 trans-membrane domain that are critical for voltage sensing (Santi et al., 2006). As a consequence, SLO2 family channels lack the property of acute voltage sensitivity found in many voltage-activated ion channels carrying these conserved positive charges. Considering the weak voltage sensitivity of SLO2 family channels, we reasoned that these channels might be controlled in ways in addition to voltage and investigated the possibility of their being controlled by metabotropic neuromodulators (Santi et al., 2006). In that study, we showed that SLO2 family channels expressed in *Xenopus* oocytes can be modulated by several neuromodulators acting through coexpressed G protein–coupled receptors (GPCRs). Because those experiments were undertaken in a heterologous expression system coexpressing SLO2 channels and GPCRs from different sources, and not native neurons (Santi et al., 2006), we commenced a study to determine which GPCRs might be present in native rat mitral cells, which are known to express high levels of sodium-activated potassium channels (Egan et al., 1992a, b; Bhattacharjee and Kaczmarek, 2005). Thus, we undertook a series of immunocytology experiments as well as gleaned relevant information from the Allen Brain Atlas and the literature. These studies revealed that GABA-B GPCRs are expressed at a conspicuously high density on the soma of mitral cells (Fig. 2*B, E*). We also showed that GABA-B receptors are coexpressed with the Na^+^-activated K^+^ channel, SLO2.1, as well as voltage-gated TTX-sensitive sodium channels known to conduct both persistent and transient components of the sodium current (Fig. 2*D, G*). Because of the abundant presence of GABA-B receptors in a cell type known to have robust expression of SLO2.1 channels (Panzanelli et al., 2004), we undertook experiments to investigate whether the IK_Na_ current could be modulated by GABA-B receptors. Remarkably, application of baclofen, a specific GABA-B activator (Bowery, 2006), to voltage-clamped rat mitral cells produced a change in whole-cell currents that is virtually identical to the change produced by TTX application. As shown in Fig 1*B*, a large component of delayed non-inactivating outward current was removed after application of 10 µM baclofen [the outward current reduction at 40 mV was 30.4 ± 2.5% (*n* = 12, *p* < 0.0001)], along with what appeared to be the persistent sodium current (Fig. 1*B*, bottom panel, arrow). Thus, activation of GABA-B receptors appear to inhibit the coupled system of INa_P_ and IK_Na_ in a manner highly similar to that of TTX, where the primary effect appears to be on the inhibition of the persistent sodium current, which leads to a secondary effect, the inhibition of the sodium-dependent outward current.

### Baclofen and GABA evoke similar effects

As a further check to verify that the observed action of baclofen is on GABA-B receptors and not due to an off-target effect, we examined the effect of GABA on whole-cell currents of rat mitral cells. Whereas baclofen is highly selective for GABA-B receptors, GABA activates both ionotropic GABA-A and GABA-B receptors. Nevertheless, we should be able to duplicate the effect of baclofen in this experiment if we can subtract out any current components elicited through GABA-A, while viewing the remaining GABA-B effects. The first notable effect of adding GABA was the appearance of a large membrane leak component, ostensibly due to the activation of GABA-A receptors. The subtraction of the GABA-A current component turned out to be possible because it resembled a linear steady-state leak component. Thus, after subtracting this large leak component from the recorded current, the remaining current and its subtracted component were remarkably similar to that seen after the application of baclofen (Fig. 1*C*). After application of 100 µm GABA, the outward current reduction at 40 mV was 22.9 ± 2.1% (*n* = 5, *p* < 0.0001). This result provides further support that the effect of baclofen on the whole-cell current of mitral cells is specifically through the GABA-B receptors. One-way ANOVA was performed on the effects of TTX, baclofen, and GABA, and no significant difference was found (*p* > 0.05).

### GABA-B activation targets the persistent sodium current

**Figure 3.**
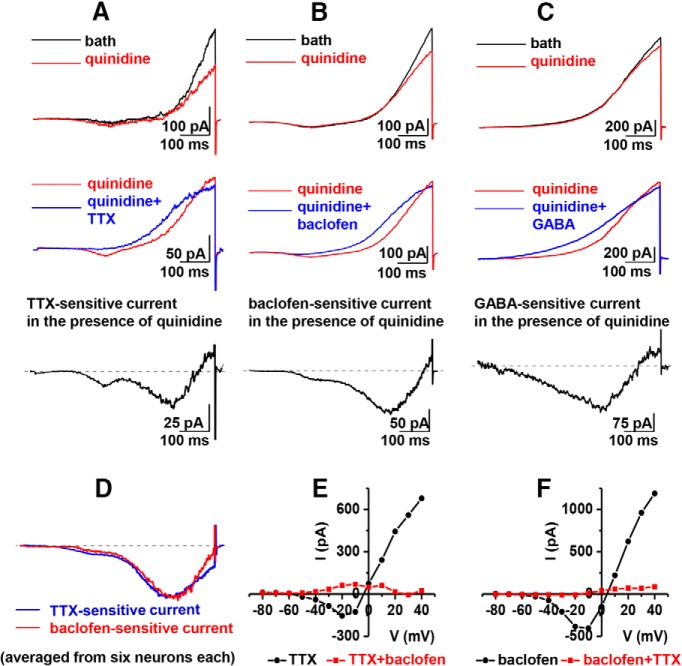
Similar persistent inward currents are inhibited by TTX, baclofen, and GABA. The top panels show examples of whole-cell ramp currents recorded from cultured rat mitral cells in the absence (black) and presence (red) of 20 µM quinidine. A slow ramp protocol from a holding potential of –70 mV to 10 mV in 600 ms was applied. The blue traces shown in the middle panel are the ramp currents recorded from the same cells after addition of 0.2 µM TTX (***A***), 10 µM baclofen (***B***), or 100 µM GABA (***C***). The traces below show the inward current components removed by TTX (***A***), baclofen (***B***), and GABA (***C***) obtained by subtraction of blue from red traces in the panels above. Na^+^ and Ca^2+^ were absent in the intracellular pipette solution, and Ca^2+^ was absent in the extracellular recording solutions. ***D***, Averaged TTX-sensitive (blue) and baclofen-sensitive (red) ramp current traces from normalized drug-sensitive traces are similar. The averaged TTX-sensitive ramp current reaches a peak value of –181 ± 40 pA at –10.4 ± 4.3 mV (*n* = 6). The averaged baclofen-sensitive ramp current reaches a peak value of –193 ± 77 pA at –15.2 ± 5.1 mV (*n* = 6). ***E, F***, Prior treatment with TTX does eliminate the effect of baclofen and *vice versa*, suggesting that both agents eliminate the same current. Representative I-V plots show that neither inward sodium current nor outward potassium current is further reduced by the other agent after exposure to one of the agents. Cultured rat mitral cells were first treated with 0.2 µM TTX or 10 µM baclofen for 10 min, then with coapplication of 0.2 µM TTX or 10 µM baclofen, respectively, for another 5 min. Application of 10 µM baclofen in the presence of 0.2 µM TTX does not significantly reduce outward current (4.3 ± 2.1%, *n* = 4, *p* > 0.05), nor does coapplication of 0.2 µM TTX in the presence of 10 µM baclofen (2.2 ± 1.0%, *n* = 5, *p* > 0.05).

Because the results with baclofen so closely mirrored the results with TTX, we reasoned that the direct effect of baclofen was to eliminate the persistent sodium current, whereas a secondary indirect effect was to eliminate the sodium-dependent outward current. To more clearly observe this hypothesized primary effect of baclofen on the persistent sodium current, we used a slow-ramp voltage-clamp protocol to directly observe the persistent sodium current uncontaminated by the transient component of sodium current (Estacion and Waxman, 2013). In addition, we applied quinidine, a K^+^ channel blocker, to remove a portion of outward current to more clearly reveal the persistent sodium current. The results are shown in Fig. 3. Using this slow-ramp protocol in the presence of quinidine, we examined the effects of TTX, baclofen, or GABA on the inward current component from rat mitral cells. As shown in Fig. 3*C*, the inward current component is sensitive to each of these three agents and is remarkably similar in various properties including amplitude, kinetics, and voltage range of activation. Note that the earlier and smaller of two components of inward current present in some traces may be an early “window” component that is dependent on closed-state inactivation. The second and larger component at more depolarized potentials is the persistent sodium current that is detected for many tens of milliseconds after the start of a depolarizing pulse (Estacion and Waxman, 2013). To further illustrate the similarity between the inward currents eliminated by TTX and baclofen, we averaged together the persistent currents obtained from six neurons exposed to TTX and six exposed to baclofen and overlapped the traces as shown in Fig. 3*D*. The virtual identity of the two currents compared in this way is striking (see legend for quantitative data). It is known that more than one voltage-gated sodium channel (VGSC) type is expressed in olfactory mitral cells (Krzemien et al., 2000; Schaller and Caldwell, 2000), and most VGSCs can express small amounts of persistent sodium current. Although it is conceivable that their sensitivities to either TTX or baclofen may differ, the similarities of the overlapped traces shown in Fig. 3*D* suggest that the same current components are being removed by both agents. Prior treatment with TTX effectively eliminates the effect of baclofen (Fig. 3*E*, 4.3 ± 2.1%, *n* = 4, *p* > 0.05) and *vice versa* (Fig. 3*F*, 2.2 ± 1.0%, *n* = 5, *p* > 0.05), also indicating that both agents reduce the same current component. In a previous study observing single-channel sodium currents in mitral cells, we showed that the TTX-sensitive persistent current in these cells was due to the reopenings of voltage-sensitive sodium channels after the inactivation of the fast transient sodium current component (Hage and Salkoff, 2012). Thus, like the effect of TTX on these neurons in which the elimination of the outward component of current is a secondary effect caused by the primary effect of eliminating the persistent sodium current (Budelli et al., 2009; Hage and Salkoff, 2012), the neuromodulatory effect of GABA-B stimulation appears to primarily be reducing the persistent sodium current. As will be discussed, we presume that TTX directly interacts with and blocks the sodium channels, whereas baclofen or GABA acts though the GPCR signaling cascade to block the sodium channels.

### Baclofen has no direct effect on IK_Na_

**Figure 4.**
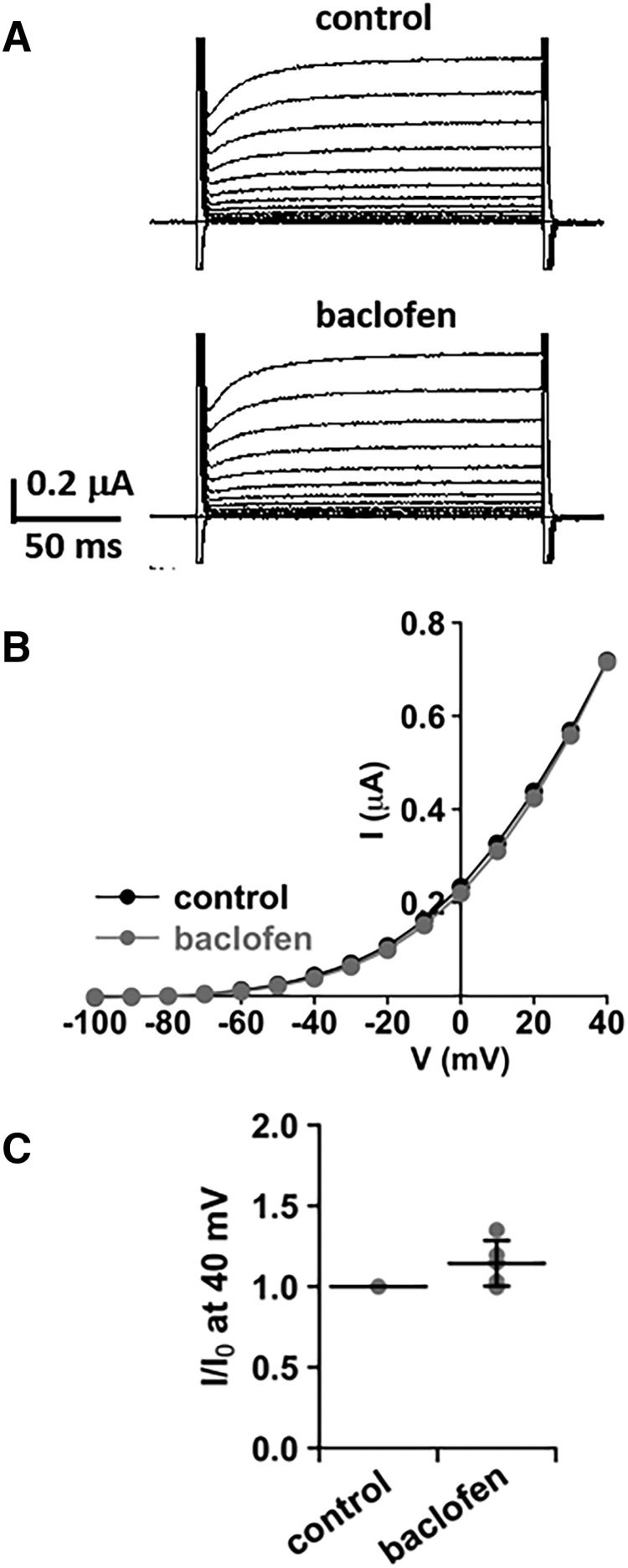
Baclofen has no direct effect on SLO2.1 channels. ***A***, Whole-cell currents of SLO2.1 channels expressed in *Xenopus* oocytes before (control) and after the addition of 10 μM baclofen. The currents were evoked by voltage pulses from –100 to 40 mV in 10-mV steps at a holding potential of –80 mV. ***B***, Representative I-V plot of currents from ***A*** shows that baclofen has no direct effect on SLO2.1 currents. Plot in ***C*** summarizes the current measurements at 40 mV before (control) and after baclofen treatment. Current values were normalized by control. Mean value (black horizontal line) and SD error bar are shown. There is no significant change in current amplitude after baclofen treatment (*n* = 5, *p* > 0.05).

Fig. 4*A* shows an example of whole-cell currents of SLO2.1 channels expressed in *Xenopus* oocytes before (control) and after (baclofen) the addition of baclofen. After application of 10 µm baclofen, the outward current at 40 mV was unchanged compared with the control (Fig. 4*B*, *C*, *n* = 5, *p* > 0.05). These data further support that inhibition of IK_Na_ by baclofen is secondary to the suppression of persistent sodium current, and that baclofen has no direct effect on IK_Na_.

## Discussion

### What is the functional significance of GABA-B modulation of the INa_P_/IK_Na_ system?

The absence of a canonical voltage sensor in the SLO2 channels that carry the sodium-activated K^+^ current results in channels that have only weak voltage sensitivity and therefore can open and participate in membrane electrical events over a wide voltage range. These channels are actively expressed in the cell bodies of neurons from all over the brain (Egan et al., 1992a). The cell bodies of mitral cells of the olfactory bulb are packed with sodium-activated potassium channels (100% of patches pulled from the cell body have K_Na_ channels) and appear to be a major determinant of the intrinsic excitability of these cells. As in many mammalian neurons, the cell body is interposed between the distal dendritic inputs (the “tufts” in mitral cells) and the axon initial segment, which is a site of action potential initiation and therefore the output of the cell. If all K_Na_ channels in the cell body were constitutively active, incoming synaptic potentials would easily be shunted and greatly diminished by the high K^+^ conductance of the soma, and never reach the axon spike initiation zone. This strategic placement of the channels could be one plausible reason for a mechanism of neuromodulation of the channels. Thus, if K_Na_ channels are down-regulated by metabotropic signaling at the soma in mitral and other output neurons, integrated synaptic potentials would be able to pass through the cell body to the spike initiation zone and generate an outgoing action potential. Hence, GABA acting on GABA-B receptors, which in turn act on voltage-gated sodium channels, which in turn act on sodium-activated potassium channels (all abundantly expressed on mitral cell bodies), would serve to modulate synaptic effectiveness in this cell type. Additionally, however, GABA signaling through GABA-A receptors as well as other GABA-B mechanisms of action are undoubtedly present in the mammalian olfactory bulb and appear to be involved in complex dendrodendritic interactions undergone by the lateral dendrites of mitral cells and the dendrites of interacting granule cells (Isaacson and Vitten, 2003). However, it might be noted in this context that GABA-B receptors are more sensitive to endogenous GABA compared with GABA-A (Sodickson and Bean, 1996).

### Multifaceted neuromodulatory control of K_Na_ channels

The neuromodulatory control of K_Na_ channels is apparently multifaceted and complex. It was shown in a heterologous expression system that K_Na_ channels can be directly modulated by metabotropic signaling through the G-α_*q*_ signaling pathway (Santi et al., 2006). However, the coupling of the activity of K_Na_ channels to the persistent sodium current creates additional possibilities for modulating K_Na_ channels, and this appears to be the mechanism by which the GABA-B system modulates it. A previous report of sodium channel modulation by direct G protein βγ activity shows an enhancement of persistent sodium current rather than a diminution (Mantegazza et al., 2005). Our findings, however, suggest a neuromodulatory pathway of downregulation of the persistent sodium current. GABA-B is reported to signal via the Gi-coupled GPCR pathway (Bettler et al., 2004). However, the signaling pathways of GABA-B receptors are complex and not yet fully resolved. GABA-B signaling pathways are known to be highly variable and are known to interact with a variety of other signaling pathways (Thompson and Gahwiler, 1992; Neves et al., 2002; Karls and Mynlieff, 2015). Thus the mechanism of GABA-B signaling to reduce persistent sodium current remains to be investigated. Nonetheless, our finding—a functional relationship between widely distributed GABA-B receptors (Prosser et al., 2001; Schuler et al., 2001; Bettler et al., 2004) and widely distributed sodium-activated potassium channels (Egan et al., 1992a, b; Bhattacharjee and Kaczmarek, 2005)—suggests that we may have revealed a significant functional mechanism for GABA-B neuromodulation.

### Does the effect of GABA-B activation have a net positive or inhibitory effect on excitability?

The interrelatedness of INa_P_ and IK_Na_ suggests new ways that neurons may tune their excitability, and our finding that this system is controlled through the GABA-B pathway presents new insights for controlling cell excitability through GABA-B modulators. Whether these two interacting currents have a net excitatory or inhibitory effect depends on the amplitudes and balance of the two currents in a particular neuronal type. INa_P_/IK_Na_ coupling is a negative feedback system; INa_P_ is excitatory and IK_Na_ is inhibitory to cell electrical activity. GABA-B activation could have a net excitatory effect in neurons such as mitral cells in which IK_Na_ is a large component of the outward current; inhibition of a small inward persistent sodium current may inhibit a much larger potassium current, producing a net inhibitory effect.

Currently the only FDA-approved drug to target (activate) the GABA-B receptor is baclofen, which is used to treat muscle spasticity disorders. However, baclofen has many undesirable side effects that could arise from an unintentional excitatory effect (Vergnes et al., 1997; Bowery, 2006). Nevertheless, GABA-B activators and inhibitors could be highly useful agents to treat many neurologic disorders once a more detailed understanding of the physiologic and molecular basis of GABA-B signaling and downstream effector molecules is acquired. It is hoped that the work presented here will aid in the understanding and design of GABA-B modulators that could be helpful and resourceful pharmacological agents.

## Synthesis

Reviewing Editor: Lorna Role, Stony Brook Decisions are customarily a result of the Reviewing Editor and the peer reviewers coming together and discussing their recommendations until a consensus is reached. When revisions are invited, a fact-based synthesis statement explaining their decision and outlining what is needed to prepare a revision will be listed below. The following reviewer(s) agreed to reveal their identity: David Mott.

Overall evaluation:

Li and colleagues, use whole cell recording and immunofluorescence to investigate GABAB receptor modulation of INaP and IKNa in cultured rat mitral cells of the olfactory bulb. This work builds on prior reports from this group showing that INaP activates IKNa in mitral cells.

The main findings are that GABAB receptors are co-localized with SLO2.1 and NaV1.6 on mitral cells and that activation of these GABAB receptors inhibits both INaP and IKNa.

The authors conclude that the primary effect of GABAB receptor activation is to inhibit INaP and that inhibition of the coupled IKNa is secondary to the suppression of INaP. They suggest that by reducing IKNa, GABAB receptors may act as a 'gatekeeper' of synaptic potentials in mitral cells.

This study includes novel observations and was conducted using appropriate methodologies. Despite the limited scope of the work, the report does offer a better understanding of the contribution of GABAB receptors to neuronal function.

The reviewers and editor conferred and outlined a set of “must-do” revisions that are the minimum required for consideration for publication of the study by eNeuro. In addition, we have compiled a set of additional suggestions and experiments that the authors might want to do to further the impact of their manuscript.

Must do revisions for re review:

1. Authors should adopt a more conservative tone in the discussion as the study lacks demonstration of functional impact in intact circuit.

The scope of the experiments done in this study is limited in that it does not include direct demonstration of the functional impact of the GABA B modulation on mitral cells within an intact circuit. As these experiments would be an extensive addition to the current work (see below: suggested studies), the reviewers ask instead that in the presentation and in particular in the discussion of the work, the authors are a bit more measured in their interpretations. Specifically, the authors' discussion of the role of the GABAB modulation in regulating excitability of mitral cells and the proposal of a 'gatekeeper' function seems overly speculative.

2. The authors need to incorporate (into the current manuscript) all additional data that has been collected and is referred to in the rebuttal letter from the JNS reviews.

There are several points that were raised in prior reviews that were mentioned in the rebuttal letter that were not included in the manuscript. All of these data should be presented in figure form, in the text of the Results and taken into consideration in the discussion. These include:

a. The authors must include the data that show that GABAB does not directly affect IKNa (i.e. their data that baclofen does not directly affect IKNa ) to support their claim that the effects of GABAB receptors in their system on IKNa are indirect, through INaP. These data are mentioned in the rebuttal letter but not included in the manuscript.

b. The authors indicate in the rebuttal that they have conducted the peptide competition control experiments for two of their antibodes, for SLO2.1 and NaV1.6. They observed essentially complete abolishment. The data pertinent to this should be shown and at least a sentence to this effect should be added to the Methods ( we were not able to find one).

c. The authors must add the statistics to and clarify the issues in the graphs represented in Figs 3E and F . Specifically, the authors state in the rebuttal letter that they have now performed statistical analysis that shows that the effects of baclofen and GABA on the whole cell current in Fig. 1 are not different from each other (p > 0.05). This is however not presented in the paper and it should be. The lack of difference is not obvious from the mean {plus minus}SE values given in the legend.

Additional suggested work that would improve the impact of the work:

a. The manuscript would be strengthened by additional occlusion experiments showing that TTX, baclofen and GABA occlude each other's action on INaP.

b. The manuscript would be greatly strengthened by direct demonstration of the functional impact of the novel GABAB modulation. Specifically, the focus of thie current manuscript is on GABAB receptor-mediated inhibition of INaP and IKNa in cultured mitral cells.

c. Based on the data presented, it is unclear what, if any, net effect this action of GABAB receptors would have on neuronal excitability in olfactory bulb. Experiments in brain slices showing the effect of GABAB receptors on the excitability of mitral cells would strengthen this work.
